# Statistical Modeling of Seafood Fraud Highlights Uncertainties in Products From Metro Vancouver, British Columbia, Canada: Revisiting Hu et al. (2018)

**DOI:** 10.1111/1750-3841.71201

**Published:** 2026-06-18

**Authors:** Jarrett D. Phillips, Fynn A. De Vuono‐Fraser

**Affiliations:** ^1^ School of Computer Science University of Guelph Guelph Canada; ^2^ Department of Integrative Biology University of Guelph Guelph Canada; ^3^ Department of Mathematics and Statistics University of Guelph Guelph Canada

**Keywords:** Bayesian inference, frequentist inference, generalized linear models (GLMs), Hamiltonian Monte Carlo (HMC), logistic regression, seafood mislabeling rates

## Abstract

Seafood misrepresentation, encompassing product adulteration, mislabeling, and substitution, among other fraudulent practices, has risen globally over the past decade, greatly impacting both the loss of important fish species and the behavior of human consumers alike. While much effort has been spent attempting to localize the extent of seafood mislabeling within the supply chain, strong associations likely existing among key players have prevented timely management and swift action within Canada and the United States in comparison to European nations. To better address these shortcomings, herein frequentist and Bayesian logistic generalized linear models (GLMs) are developed in R and Stan for estimation, prediction, and classification of finfish product mislabeling in Metro Vancouver, British Columbia, Canada based on product source (grocery store/restaurant/sushi bar), state (cooked/raw), appearance (modified/plain), form (chopped/chunk/fillet/whole), and color (light/red). Obtained results based on odds ratios and probabilities for 281 seafood samples from 92 grocery stores, 82 restaurants, and 107 sushi bars paint a grim picture and are consistent with general trends found in past studies, especially when products have been altered. In addition to estimation and prediction, analyses are extended through applying regression models to classify four collected seafood samples whose labeling status (correctly labeled or mislabeled) was not known a priori. While all samples were deemed correctly labeled, point and confidence/credible intervals differed markedly, indicating considerable uncertainty as to true mislabeling rates. This work paves the way to rapidly assess the current state of knowledge surrounding seafood fraud nationally and on a global scale using established pedagogical statistical methodology.

**Practical Applications:**

Seafood is one of the largest global food commodities but also among the most frequently subject to fraud, including substitution, adulteration, and mislabeling to evade tariffs or conceal illegal, unreported, and unregulated (IUU) fishing. Consumers often cannot verify what they purchase, especially when identifying features like skin, fins, and scales are removed or products are heavily processed through cooking or canning. Despite its prevalence, the timing, location, and mechanisms of seafood fraud within supply chains remain poorly understood. This study applies statistical regression modeling to investigate and address these uncertainties.

## 1 Introduction

Seafood fraud is an escalating problem impacting society today, where both economic loss and substantial harm to fish populations have been sorely felt, especially in Canada and the United States (Cawthorn and Mariani 2017; Cawthorn et al. 2018, 2021; Hanner et al. 2011; Hu et al. 2018; Naaum and Hanner 2015; Naaum et al. 2015; Shehata, Bourque, et al. 2019; Shehata et al. 2018; Warner et al. 2019). In its simplest form, seafood fraud is the practice of misleading consumers about products to increase profits, through adulteration, mislabeling, and substitution, among other suspect behaviors (Naaum et al. 2016). Canada is not exempt in this respect, and is well above the global average mislabeling rate of 30% found by Pardo et al. (2016) in an examination of 51 published studies targeting 4500 samples. One aspect of food fraud pertaining especially to the Canadian market in comparison to countries within Europe, where specific labeling laws and up‐to‐date and comparable regulatory seafood lists are more tightly regulated (Cawthorn and Mariani 2017; Cawthorn et al. 2018, 2021), is seafood product mislabeling. Unfortunately, Canada and the United States have a unique problem where labeling policies, gleaned through the Canadian Food Inspection Agency (CFIA) Fish List and the United States Food and Drug Administration (USFDA) Seafood List, respectively, are extremely vague. This has led to the compounding of seafood mislabeling rates at all levels of the supply chain both outside of and within Canada (Shehata, Bourque, et al. 2019). For example, Canadian import guidelines allow over 200 species to be labeled and sold as snapper, a group which includes vulnerable species such as the Northern red snapper (*Lutjanus campechanus*). In a recent (and so far only) meta‐analysis of seafood mislabeling (comprising species substitution and conflicting and unacceptable market names) within the United States (32 states) targeting 35 studies between 2010 and 2023, an overall mislabeling rate of 39.100% across 4179 collected samples was found (Ahles et al. 2025), with 18.200% of samples traced to the latter two mislabeling types mentioned above. When considering only the top 10 most consumed species, while these figures are reduced, they are still alarmingly high (Ahles et al. 2025). Typically, determination of species‐level mislabeling is achieved on the basis of DNA‐based specimen identification techniques such as DNA barcoding (Hebert, Cywinska, et al. 2003; Hebert, Ratnasingham, 2003). Estimates suggest mislabeling rates of 50% and higher throughout the supply chain, but reliability largely depends on the maturity of genomic reference sequence databases, such as the Barcode of Life Data Systems (BOLD) (Ratnasingham and Hebert 2007), upon which confident species‐level matches can be readily ascertained from high‐quality taxon records (Phillips et al. 2023). This, in turn, is strongly driven by levels of standing intraspecific haplotype variation and the presence of sufficiently wide DNA barcode gaps separating within‐ versus among‐species marker diversity (Phillips et al. 2019, 2022). To ensure DNA sequence databases are populated with statistically defensible data, algorithms like HACSim (Phillips et al. 2020) should be employed to estimate required specimen sample sizes needed to capture desired levels of existing species genetic variation. Wong and Hanner (2008) were among the first to assess the efficacy of barcode reference libraries in identifying mislabeled seafood collected from markets across Canada and the United States. Out of the 91 obtained samples that successfully amplified, 25.275% (23/91) were found to be mislabeled (Wong and Hanner 2008). Despite high fraud levels within the seafood supply chain, the spatiotemporal parameters of consumer product labeling remain obscure, but are not isolated to a single source (Shehata, Bourque, et al. 2019). Species mislabeling manifests at all times of year and involves many key participants within the supply chain hierarchy, including fishers and retailers. Strong correlations also likely exist with species stock abundance, market price, and conservation status, hindering reliable fraud prediction.

The extent to which seafood products are mislabeled depends on a number of factors, such as species biomass availability, market price, and conservation status. Further, product adulteration can be accidental and/or deliberate and can occur across different stages of seafood production, processing, and sale. Nontrivial differences in prices between wholesale‐ and retail‐purchased seafood has increased opportunities for market fraud. Improving access to provenance history in the form of chain of custody documents will ensure transparency of seafood is not compromised. A widespread pattern in the seafood supply chain is the substitution of species of higher market value with those of lower market value in order to increase profitability by deceiving buyers and bypassing tariffs on product import (Donlan and Luque 2019). High‐cost species are often of conservation concern due to decreasing population numbers as a result of overharvesting fueled by rising consumer demand, and thus are often substituted with highly abundant low‐cost species (Zander and Feucht 2018). Seafood mislabeling is also exercised as a means to conceal at‐risk species, such as sharks, sourced from Illegal Unreported and Unregulated (IUU) fisheries for the sale of shark fins within the Asian market (Hanner et al. 2016). Instances of mislabeling can also pose serious health risks (Shehata, Naaum et al. 2020) or challenge established religious doctrines and lifestyle choices. For instance, overconsumption of escolar (*Lepidocybium flavobrunneum*), which is often misrepresented as butterfish or white (Albacore) tuna, can lead to serious gastrointestinal issues along with other symptoms (Pardo et al. 2016). As a result, the sale of escolar is banned in many countries such as Italy, Japan, and South Korea. All the abovementioned behaviors seem to suggest that seafood fraud is intentional, as seafood product mislabeling in the Canadian economy appears to have risen in the past decade, negatively impacting the environmental sustainability of fish stocks. Oceana Canada, a not‐for‐profit charitable ocean conservation organization subsidiary of the larger Oceana, has conducted numerous national surveys highlighting the detrimental impacts of seafood mislabeling across several major Canadian metropolitan cities in recent years. Findings are unsurprising: their 2018 survey indicated an overall mislabeling rate of 44% across nearly 400 samples and five large cities (Halifax, Ottawa, Toronto, Vancouver, and Victoria), with Victoria having the highest mislabeling average at 67%, and snapper being among the most frauded species (Oceana 2018). This trend appears to be on the rise as evidenced from a more recent survey conducted in 2021 which saw a 47% mislabeling rate across 94 samples collected from four metropolitan cities (Halifax, Montreal, Ottawa, and Toronto) (Oceana 2021).

While there have been hundreds of studies published on seafood fraud over the past decade, using basic statistical approaches, such as chi‐square tests (e.g., Shehata, Bourque, et al. 2019; Ahles et al. 2025), the majority of research has simply demonstrated that mislabeling is evident and still largely persists within the supply chain on a global scale. Despite this, a handful of publications have attempted, with some degree of success, to assess the extent of economically motivated fraud through the quantification of overall mislabeling rates at various scales globally, particularly through meta‐analyses (e.g., Stawitz et al. 2016; Luque and Donlan 2019; Giusti et al. 2023; Ahles et al. 2025). Unfortunately, many do not report measures of uncertainty around estimates such as standard errors (SEs) or confidence intervals (CIs). Overall mislabeling rates can be assumed to approximately follow a Binomial distribution as individual seafood product samples are either correctly labeled, or not. That is, each collected sample can be considered an independent Bernoulli trial governed by binary success and failure outcomes, similar to a coin toss (heads/tails), yes/no, or true/false dichotomies, for instance. Thus, a naïve way forward in this regard is to calculate the estimated Wald SE of the sample proportion of mislabeled samples, which is given by

(1) $$\hat{SE[\hat{p}}]=\sqrt{\frac{\hat{p}\left(1-\hat{p}\right)}{n}}$$

where $\hat{p}=\frac{X}{n}$ is the maximum likelihood estimator (MLE) of the population mislabeling rate, X is the number of mislabeled observations, and n is the number of samples. However, the above formula is problematic for several reasons. First, Equation (1) is a Normal approximation based on large‐sample theory through the central limit theorem (CLT); thus, large sample sizes are required for reliable estimation. When few observations are available, SEs will be large and inaccurate, leading to low statistical power. Further, resulting interval estimates could span values less than 0 or greater than 1, or have 0 width which is practically meaningless. Second, when mislabeling rates are exactly 0% or 100%, SEs will be exactly 0%. For species like snapper, where 100% mislabeling rates are often found, the above equation is not useful. In terms of sample collection, market survey designs currently lack statistical rigor, which generally manifests as convenience sampling along with pseudoreplication, as only the most commonly frauded species are targeted within mislabeling studies. For example, many fish fillets purchased in supermarkets can be traced back to a single supplier. From a statistical standpoint, this means that samples are not independent of one another, since seafood products available at retail outlets, for instance, do not constitute a simple random sample. Therefore, more sophisticated approaches are warranted.

In an effort to place seafood fraud on more statistical footing through analyzing existing data from past work, here, generalized linear models (GLMs) within the frequentist and Bayesian inference paradigms are employed to develop a logistic regression model to estimate seafood product mislabeling rates using predictors known to be informative in the detection of fraud within the Canadian supply chain, in particular, the source (outlet) of obtained seafood samples, in addition to the level of product processing which alters the physical properties of seafood. A case study conducted by Hu et al. (2018) on mislabeling of finfish products in Metro Vancouver is employed as an illustration to show the overall utility of a rigorous statistical framework for mislabeling estimation, prediction, and classification. Statistical regression modeling of seafood fraud data for the characterization of product mislabeling rates is limited within the primary literature. As such, throughout, a methodological, pedagogical, and pedantic approach is taken to highlight the nuances and assumptions inherent in both frequentist and Bayesian methods, ensuring that readers understand not only the practical implications of these models, but also their underlying statistical principles and limitations. Special attention is given to model selection, diagnostic checks, and the interpretation of results, with the goal of fostering a comprehensive understanding of how these techniques can be applied to real‐world issues in seafood fraud detection. This approach is necessary given that much statistical advice and recommendations available through online resources and elsewhere, while plentiful, are often scattered, conflicting, and contradictory, depending on the source. While researchers in food science may be familiar with many frequentist and Bayesian inference techniques, they may be less acquainted with the breadth of computational software tools available to them, particularly as models become more complex and require greater scrutiny in development, testing, and interpretation. By examining these methods in detail, the present study aimed to contribute to the development of more reliable and robust statistical tools for combating seafood mislabeling, while also offering a transparent framework that can be adapted to similar fraud detection challenges in other food sectors, such as meat (Naaum et al. 2018; Shehata, Naaum, et al. 2019).

## 2 Case Study: Uncovering Seafood Mislabeling Trends in Metro Vancouver, British Columbia, Canada

### 2.1 The Dataset

Within Hu et al. (2018), a total of 285 finfish samples was collected randomly or based on product availability from various grocery stores (G; n = 93), restaurants (R; n = 84), and sushi bars (S; n = 108) across Metro Vancouver (comprising Vancouver, Burnaby, Coquitlam, Surrey, Langley, and North Vancouver, among others) from September 2017 to February 2018. For samples purchased as takeout from restaurants and sushi bars, taxonomic identity was verified through consultation with establishment employees. Samples were subjected to polymerase chain reaction (PCR) amplification and DNA sequencing using standard laboratory protocols (Hu et al. 2018). Obtained DNA barcode sequences (Hebert, Cywinska, et al. 2003; Hebert, Ratnasingham, et al.^2003^) (272 full length 652 basepair barcodes and nine minibarcodes) were then matched to known references found within BOLD(http://www.boldsystems.org) (Ratnasingham and Hebert 2007). Specimen identifications of product samples gleaned from DNA barcoding were taken at face value even though marker length variation is expected to affect taxonomic resolution depending on the primers utilized for sequence amplification, or the level of sample processing applied after catch (e.g., cooking/canning). For instance, in Wong and Hanner (2008), species mislabeling was based on a DNA sequence similarity top match threshold of 97%. That is, barcodes were deemed to originate from different species whenever sequences from samples found in seafood markets differed by at least 3% from those in BOLD or GenBank (https://www.ncbi.nlm.nih.gov/genbank/). Because labeling status was missing for four samples (G80, R2, R51, and S10), as they failed to yield either a full length or mini DNA barcode sequence using Sanger sequencing (Hu et al. 2018), these data were excluded from the model and instead utilized for downstream prediction and classification (Table 1); see Section 3.2.3 for further details. From a statistical standpoint, these four samples were likely missing not at random (MNAR), that is, the probability of missingness depends on unobserved or underlying properties tied to the missing data themselves. Possible reasons for PCR amplification failure of the seafood product samples noted by Hu et al. (2018) include low DNA yield due to sample degradation, the presence of PCR inhibitors, such as lipids within the samples, or the inadequacy of Sanger sequencing, necessitating the use of techniques like quantitative PCR (qPCR) or high‐throughput sequencing (HTS). However, it is impossible to know the exact cause with full certainty.

**TABLE 1:** Characteristics of seafood samples with unknown labeling status.

**Source/sample**	**State**	**Appearance**	**Form**	**Color**
G80	Raw	Modified	Fillet	Light
R2	Cooked	Modified	Fillet	Red
R51	Cooked	Modified	Fillet	Light
S10	Raw	Plain	Fillet	Light

While data exclusion is often frowned upon, since it can lead to a loss of statistical power to detect real effects and potential estimation bias, missing data accounted for only 1.404% of the dataset. Thus, 281 samples (representing 98.596% of the entire dataset) were employed for all subsequent analyses (see Tables S1–S5 for a breakdown by labeling status [i.e., correctly labeled or mislabeled]). Collected samples were assessed based on the following characteristics: source (grocery [n = 92], restaurant [n = 82], and sushi bar [n = 107]), state (cooked [n = 87] and raw [n = 194]), appearance (modified [seasoned, or covered with breading/other ingredients; n = 124] or plain fillet [n = 157]), product form (chopped [n = 3], chunk [n = 27], fillet [n = 244], and whole [n = 7]), and color of the flesh (light [i.e., pink/white; n = 177] or dark [i.e., red; n = 104]).

Based on Table 2 in Hu et al. (2018), 57 (81.429%) mislabeled seafood products originated from Vancouver, with all mislabeled sushi samples (n = 24) being procured from therein. Findings by Hu et al. (2018) revealed an overall average mislabeling rate of $\hat{p}$ = 24.911% (70/281; $\hat{SE[\hat{p}}]$ = 0.026 by Equation (1); 29.268% [24/82] in restaurants, 23.913% [22/92] in grocery stores, and 22.430% [24/107] in sushi bars). Three species possessed the highest mislabeling rates: snapper (31/34; 91.176%), yellowtail tuna (5/6; 83.333%), and sea bass (3/6; 50.000%); all haddock (n = 4), mackerel (n = 16), swordfish (n = 4), and sablefish (n = 7) products were correctly labeled (Hu et al. 2018). In addition, Hu et al. (2018) implemented a binary logistic GLM in MATLAB (The MathWorks Inc. 2022) using all surveyed covariates as predictors to assess mislabeling of collected seafood products. Obtained significance test results can be found in Table 3 of their study.

### 2.2 The Model, Analysis, and Overall Findings

While Hu et al.'s (2018) study provided valuable empirical insights, several statistical aspects of the analysis warrant further examination, particularly regarding model specification and uncertainty quantification. In an effort to verify results of their GLM and validate claims made by the authors, data were refitted herein. The present work provides an alternative statistical treatment of the survey data collected by Hu et al. (2018). As with Hu et al. (2018), model interactions are not considered here due to increased model complexity and interpretability issues, despite strong interactions among predictors being conceivable. For instance, a positive Source × Appearance interaction term which is statistically significant could suggest that the effect of Source on seafood mislabeling rates is stronger when the appearance of the seafood is altered. This in turn may signal that seafood samples obtained from restaurants may be more likely to be mislabeled when their appearance is modified compared to when it is plain. Thus, the incorporation of interaction effects is left for future work. Furthermore, it is important to note that obtained seafood samples are not likely to be independent due to many factors inherent in sample collection, particularly small sample sizes for chopped, chunk, and whole form subcategories, as well as notable class imbalance between mislabeled (70 samples) and correctly labeled (211 samples) seafood products (see Supporting Information). This makes application of mixed effects/hierarchical models challenging to implement, especially in a frequentist context. Additionally, because metadata describing supplier, distributor, or retailer, alongside other provenance information were unavailable, observations were analyzed using a fixed‐effects framework under an assumption of conditional independence. (Sections 1 and 3 provide greater explanation of this issue.) Additionally, although more sophisticated and realistic extensions to Hu et al.'s (2018) model are possible and needed, adopting it here permits easy comparison with the present model investigated in the current study. The single‐intercept ($\beta_{0}$), no‐interaction logistic regression (logit) model employed herein is given by:

(2) $$\mathrm{logit}\left(\pi \right)=\log\left(\frac{\pi }{1-\pi }\right)=\beta_{0}+\sum_{i=1}^{p}\sum_{j=2}^{k_{i}}\beta_{ij}1\left(X_{i}={\mathrm{Cat}}_{j}\right)$$

where π, the success probability (here, the seafood mislabeling rate), forms the basis of the remainder of the paper. The quantity $\frac{\pi }{1-\pi }$ is the odds in favor of product mislabeling relative to a sample being correctly labeled, p is the total number of predictors, $X_{i}$ represents the ith categorical predictor, $k_{i}$ is the number of categories (levels) for the ith predictor, $\beta_{ij}$ represents the coefficient for the jth category of the ith predictor, and $1(X_{i}={\mathrm{Cat}}_{j})$ is an indicator function that takes the value 1 when $X_{i}$ is in the jth category, and 0 otherwise. Probabilities are then computed from a simple transformation:

(3) $$\begin{array}{ccc} \pi & = & P\left(y=1|x\right)={\mathrm{logit}}^{-1}\left(\beta_{0}+\sum_{i=1}^{p}\sum_{j=2}^{k_{i}}\beta_{ij}1\left(X_{i}={\mathrm{Cat}}_{j}\right)\right)\\ & = & \frac{\exp\left(\beta_{0}+\sum_{i=1}^{p}\sum_{j=2}^{k_{i}}\beta_{ij}1\left(X_{i}={\mathrm{Cat}}_{j}\right)\right)}{1+\exp\left(\beta_{0}+\sum_{i=1}^{p}\sum_{j=2}^{k_{i}}\beta_{ij}1\left(X_{i}={\mathrm{Cat}}_{j}\right)\right)}, \end{array}$$

which makes results more easily interpretable. Specifically, the model comprised fixed effects for both intercept and regression coefficients; however, each factor level associated with a given factor (e.g., grocery, restaurant, and sushi bar in the case of Source) will not always have the same effect. Within Equations (3) and (4), a total of k = 9 parameters must be estimated (including the intercept, but excluding baseline levels for each factor, which are set to 1 by convention; see below), with p = 5 representing the source, state, appearance, form, and color of retrieved seafood samples. Thus, model parameters must be estimated numerically either through frequentist methods via iteratively reweighted least squares (IRLS) to obtain MLEs, or through Bayesian inference via the posterior distribution, each based on available data. Unlike in most standard regression scenarios, here the intercept is directly interpretable since all covariates are categorical: $\beta_{0}$ is the log odds of mislabeling when all predictors are at their reference level. By definition, reference levels correspond to $\beta_{i}=0$, giving an odds of 1 and a probability of 50%. In the context of the present case study, $\beta_{0}$ is the estimated mislabeling log odds associated with a cooked modified chopped light‐colored seafood sample collected from a grocery store. Logistic regression coefficients should be interpreted in the following way: for every 1 unit increase in a given predictor variable $x_{i}$, the log odds increases additively by $\beta_{i}$ units, given all other predictor variables are held constant. This, in turn, corresponds to a change in the odds by a multiplicative factor of $\exp(\beta_{i})$ units. While logistic regression coefficients are not straightforward to decipher, especially in the presence of categorical variables and interactions, it is important not to associate estimated effects with true causation.

Herein, in addition to estimation, probabilities obtained from model fitting are used to classify the four samples having unknown labeling status as either mislabeled (1) or correctly labeled (0) according to

$$\delta =\left\{\begin{array}{cc} 1, & \text{if}\;P(y=1|x)\geq 0.50\\ 0, & \text{otherwise.} \end{array}\right.$$

That is, samples with predicted probabilities exceeding 50% are likely to be mislabeled compared to those below this threshold, though this is just a convention. Other prediction cutoffs are possible and should be explored moving forward; a discussion is given in the Supporting Information. Since probabilities generated from logistic regression rarely equal 50% exactly, the equality above could equally apply to the correctly labeled category instead.

All analyses in the present work were carried out on a 2023 Apple MacBook Pro with M2 chip and 16 GB RAM running macOS Ventura 13.2. A random seed was set to guarantee reproducibility of obtained results. Outputted regression estimates were rounded to three decimal places of precision and interpreted as absolute percentage differences from baseline values of zero and one for log odds and odds, respectively, as well as 50% for probabilities.

#### 2.2.1 Frequentist Analysis

Since Hu et al. (2018) did not report regression estimates nor derived statistics thereof, their model was reexamined in R via glm() using the binomial family with the default logit link function, outputting log odds, odds, and probabilities, among other important quantities contained in the summary() function (Tables 2 and 3). In addition to these, associated asymmetric 95% profile likelihood CIs were also computed using the confint() function, along with monotonic transformation due to the problems inherent with symmetric Wald intervals calculated via Equation (1) discussed previously. (Note that CIs may differ slightly from run to run due to algorithm variability.) Reference levels for each factor included in the model were coded alphabetically (which is the default in R). For instance, “Grocery” served as the reference for Source since it occurs before both “Restaurant” and “Sushi bar”. In the case of L levels, there are L−1 dummy/indicator variables used to represent the categories of a factor. Typically, reference levels are designated based on the most representative factor level, or that which is most important to a given research question. However, because so little is known about the extent of seafood fraud within the supply chain, custom coding of reference levels was not utilized, but should nevertheless be explored in future work. A classic problem encountered when performing logistic regression with categorical variables in a frequentist setting is separation, which occurs when a linear combination of the data classes (here, e.g., mislabeled and correctly labeled) are perfectly predicted by underlying models (Mansournia et al. 2017). In the context of seafood mislabeling, this can occur, for example, whenever all samples belonging to a particular species have a 0% mislabeling (similarly, correctly labeled) rate or a 100% mislabeling (correspondingly, correctly labeled) rate, or when sample sizes for predictor categories are low. When this happens, parameter estimates and/or SEs will be very large or infinite due to lack of algorithm convergence. A common fix to rectify nonidentifiability in this regard is to prune predictors until the model is identifiable; however, this could result in the exclusion of highly predictive variables from the final model. Such an issue was not encountered within the study herein, as no warnings were returned by glm() during fitting, and all computed coefficient estimates and SEs exhibited numeric stability. Fortunately, the Bayesian paradigm offers a simple way forward without the need to collect additional data through techniques like model/parameter hierarchicalization, where specification of suitable priors can effectively regularize coefficients by shrinking them toward 0.

**TABLE 2:** Frequentist GLM summary results and CIs computed via maximum likelihood. The estimate column denotes the log odds relative to the reference level, whereby values are compared to a baseline of zero.

**Parameter**	**Estimate**	**Standard error**	**95% confidence interval**	**Test statistic (** *z* **)**	***p***‐value
$\beta_{0}$	2.235	1.628	(−1.303, 5.509)	1.373	0.170
$\beta_{{\mathrm{Source}}_{\mathrm{Restaurant}}}$	0.893	0.901	(−0.907, 2.692)	0.991	0.322
$\beta_{{\mathrm{Source}}_{\mathrm{SushiBar}}}$	0.061	0.390	(−0.699, 0.838)	0.156	0.876
$\beta_{{\mathrm{State}}_{\mathrm{Raw}}}$	−1.188	1.001	(−3.443, 0.598)	−1.186	0.236
$\beta_{{\mathrm{Appearance}}_{\mathrm{Plain}}}$	1.846	0.801	(0.484, 3.770)	2.304	0.021
$\beta_{{\mathrm{Form}}_{\mathrm{Chunk}}}$	−3.578	1.586	(−6.763, −0.096)	−2.256	0.024
$\beta_{{\mathrm{Form}}_{\mathrm{Fillet}}}$	−3.559	1.519	(−6.576, −0.152)	−2.343	0.019
$\beta_{{\mathrm{Form}}_{\mathrm{Whole}}}$	−1.574	1.725	(−4.942, 2.170)	−0.913	0.361
$\beta_{{\mathrm{Color}}_{\mathrm{Red}}}$	−1.740	0.411	(−2.615, −0.986)	−4.238	2.260 × $10^{-5}$

**TABLE 3:** Frequentist GLM predictor odds, probabilities, and CIs derived via the estimates column in Table 2. Odds above 1 indicate an increase in overall sample mislabeling, whereas odds below 1 signify a decrease relative to the baseline. A similar interpretation exists for probabilities at a predefined decision threshold of 50%.

**Parameter**	**Odds**	**95% confidence interval**	**Probability**	**95% confidence interval**
$\beta_{0}$	9.346	(0.272, 246.903)	0.903	(0.214, 0.996)
$\beta_{{\mathrm{Source}}_{\mathrm{Restaurant}}}$	2.443	(0.404, 14.766)	0.710	(0.288, 0.937)
$\beta_{{\mathrm{Source}}_{\mathrm{SushiBar}}}$	1.063	(0.497, 2.312)	0.515	(0.332, 0.698)
$\beta_{{\mathrm{State}}_{\mathrm{Raw}}}$	0.305	(0.032, 1.818)	0.234	(0.031, 0.645)
$\beta_{{\mathrm{Appearance}}_{\mathrm{Plain}}}$	6.332	(1.623, 43.378)	0.864	(0.619, 0.977)
$\beta_{{\mathrm{Form}}_{\mathrm{Chunk}}}$	0.028	(0.001, 0.909)	0.027	(0.001, 0.476)
$\beta_{{\mathrm{Form}}_{\mathrm{Fillet}}}$	0.028	(0.001, 0.859)	0.027	(0.001, 0.462)
$\beta_{{\mathrm{Form}}_{\mathrm{Whole}}}$	0.207	(0.007, 8.755)	0.171	(0.007, 0.897)
$\beta_{{\mathrm{Color}}_{\mathrm{Red}}}$	0.175	(0.073, 0.373)	0.149	(0.068, 0.272)

It is worth noting that SEs and CIs obtained for all estimates and parameters across factor levels are quite large and wide, signaling that robust determination of seafood mislabeling rates on the basis of product characteristics is an arduous endeavor. In comparing frequentist GLM results obtained herein to Hu et al. (2018), several findings are noteworthy. Here, only coefficients for plain appearance ($\beta_{{\mathrm{Appearance}}_{\mathrm{Plain}}}$), chunk form ($\beta_{{\mathrm{Form}}_{\mathrm{Chunk}}}$), fillet form ($\beta_{{\mathrm{Form}}_{\mathrm{Fillet}}}$), and red color ($\beta_{{\mathrm{Color}}_{\mathrm{Red}}}$) were found to be statistically significant (p≤ 0.05) (Table 2), whereas $\beta_{0}$, $\beta_{\mathrm{form}}$, and $\beta_{\mathrm{color}}$ were in the case of Hu et al. (2018). Unfortunately, since Hu et al. (2018) did not provide specific modelling details (only F‐test results were given), nor coding scripts, exact reproducibility of their findings is challenging. It is also important to note that unlike R, which is free and open‐source software, MATLAB is proprietary and subscription‐based. In particular, it is unclear what factor levels were employed by Hu et al. (2018) as references. However, like R, MATLAB uses the first level listed alphabetically to denote the reference category; thus, it is assumed that this was the case for Hu et al. (2018). The statements made by Hu et al. (2018) regarding their results are somewhat broad and may be difficult to apply in practice as statistical support provided appears limited. In addition, Hu et al. (2018) did not explain their rationale for reporting F‐test statistics and their p‐values. Within the context of logistic regression, model fit is often evaluated using deviance‐based tests; however, no regression model comparisons were reported in their analysis. The presentation in Hu et al. (2018) is at times unclear, particularly with respect to how certain comparisons were made. For example, the statement “Products composed of fish muscle of a lighter color flesh and chopped muscle tissue were more prone to illicit practices compared to red or fillet samples...” does not fully specify the comparison framework at hand, especially for product categories having more than two levels (i.e., Form in this study). Additionally, the use of broad terms such as “source” and “appearance” in Table 3 of their study raises questions about how these variables were specified in the model. Even if alternative modelling choices were adopted, differences between their results and those of the present study could still arise due to variations in implementation, including optimization algorithms, parameter settings, numerical precision, and random seed selection across software environments such as R and MATLAB. Findings herein are revealing: a cooked modified chopped light‐colored seafood sample collected from a grocery store corresponds to a 223.492% higher log odds, 834.574% increased odds, and 40.334% greater chance of being mislabeled at a threshold of 50% compared to non‐reference samples.

Further, product source ($\beta_{{\mathrm{Source}}_{\mathrm{Restaurant}}}$ and $\beta_{{\mathrm{Source}}_{\mathrm{SushiBar}}}$) may be associated with higher log odds, odds and probabilities of mislabeling relative to grocery store samples (89.308%, 144.264%, and 20.953%, and 6.103%, 6.293%, and 1.525%, respectively) (Tables 2 and 3). Similarly, $\beta_{{\mathrm{Appearance}}_{\mathrm{Plain}}}$ has a 184.559% higher log odds, 533.182% increased odds, and 36.361% greater probability at a 50% boundary of being mislabeled compared to modified products. Note that since summary() carries out a two‐sided significance test at level α (herein set at 5%) by default, there is a direct relationship between hypothesis tests and reported 95% CIs: if a hypothesized value of a population parameter (herein 0; 1; and 50% for log odds, odds, and probabilities, respectively) falls within said interval, then the null hypothesis of equality will fail to be rejected. Hu et al. (2018) point to sampling bias as a likely culprit, leading to artificially high mislabeling rates due to low numbers of collected samples. This can be seen in the Form category in the case of chopped, chunk, and whole samples (Table S4). Conversely, $\beta_{{\mathrm{State}}_{\mathrm{Raw}}}$, all Form parameters, and $\beta_{{\mathrm{Color}}_{\mathrm{Red}}}$ each have a 118.759%, 69.505%, and 26.632%; 357.802%, 97.207%, and 47.283%; 355.850%, 97.152%, and 47.231%; 157.447%, 79.288%, and 32.842%; and 174.047%, 82.456%, and 35.075% lower log odds, odds, and probabilities of mislabeling relative to cooked, chopped, and light‐colored samples, respectively.

#### 2.2.2 Bayesian Analysis

The model was then fitted using the Stan probabilistic programming language (Carpenter et al. 2017) framework with default settings for leapfrog integration (e.g., step size) to efficiently solve a system of ordinary differential equations (ODEs) via the No‐U‐Turn Sampler (NUTS) algorithm (Hoffman and Gelman 2014) for Hamiltonian Monte Carlo (HMC). The rstanarm R package (Goodrich et al. 2023) was employed to avoid needing to first compile code in the rstan R package (Stan Development Team 2023a), to ensure generation of well‐behaved Markov chains, and to aid ease of reproducibility. The same indicator variable encoding scheme used for the frequentist analysis (see previous section) was employed here.Theoretical background on HMC, as well as computational issues encountered when fitting models and how to remedy such problems, can be found in the Supporting Information for interested readers.

A variety of noninformative, weakly informative, and strong prior distributions on model parameters available in rstanarm was examined here to assess model sensitivity: (1) a N(0,2.5) prior on the intercept with non‐informative (flat) uniform U(−∞,∞) priors on regression coefficients, (2) weakly informative standard Normal N(0,1) priors on all model parameters, (3) weakly informative Normal N(0,2.5) priors on all model parameters, and (4) weakly informative Cauchy priors on all model parameters. Employment of multiple priors to assess model robustness has not been implemented in past investigations of seafood fraud such as by Luque and Donlan (2019). Prior (1) above is specified in the absence of any information regarding the true values of posterior parameters, whereas prior (2) is a common choice widely seen in the literature. Prior (3) is rstanarm's default, which tends to work well in most scenarios. The choice of prior (4) requires some explanation. The Cauchy distribution has been recommended in the context of logistic regression by several authors (Gelman et al. 2014, 2008; Ghosh et al. 2018), as well as in the Stan User Guide (Stan Development Team 2023b) and by the Stan Development Team (Stan Development Team 2020), in part due to the distribution's heavier tails compared to the Normal distribution, allowing the incorporation of outliers. In particular, Gelman et al. (2008) remark that logistic regression coefficients rarely lie outside the interval (‐5, 5) on the log‐odds scale, which corresponds to approximately (0.007, 148.413) and (0.007, 0.993) on the odds and probability scale, respectively by Equation (4). As such, a Cau(0,10) distribution is recommended on the model intercept and a Cau(0,2.5) on all other coefficients (Gelman et al. 2008). For all considered priors, model intercepts coincide with predictors centered to have mean 0. Centering in this fashion can often facilitate interpretation of models in the presence of interaction effects (Gelman 2007).

Four chains were run for 2000 iterations each in parallel across four cores. Within each chain, a total of 1000 samples was discarded as warmup (i.e., burnin) to reduce dependence on starting conditions. Further, 1000 post‐warmup draws were utilized per chain. Each of these reflects default MCMC settings in Stan. MLEs for regression coefficients given in Table 1 were used as initial values for MCMC. Alongside sample posterior means and posterior standard deviations (SDs), 95% credible intervals (CrIs) were also reported, which should be interpreted as follows, to the extent that one trusts the prior distribution selected and underlying data: Given a (1−α)100% CrI, where α is the desired significance level, the true parameter is contained within said interval with (1−α)100% probability. This is in sharp contrast to a CI, where, with repeated sampling, on average (1−α)100% of constructed intervals will contain the true parameter of interest.

Selected priors were deemed appropriate based on prior predictive checks generated in rstanarm and the bayesplot R package (Gabry et al. 2019; Gabry and Mahr 2022) to ensure simulated sample mean draws were reasonably close to observed data; the same was done for resulting posteriors (Figures S1–S8). Results in Tables 4 and 5 highlight that the Bayesian model is sensitive to the choice of prior distribution, but some parameters are affected more than others likely owing to a small number of observations for particular factor levels like those for Form. Interpretation of results in terms of percent deviation from baseline values is identical to that in Section 2.2.1. Further, for all assessed prior distributions, estimates appear close in value to their frequentist counterparts, reinforcing qualitative findings. This is especially true in the case of the uniform prior, since the posterior is completely dominated by the likelihood. Across all prior specifications considered (flat, Normal, default, and Cauchy), posterior estimates for $\beta_{{\mathrm{Source}}_{\mathrm{Restaurant}}}$, $\beta_{{\mathrm{State}}_{\mathrm{Raw}}}$, $\beta_{{\mathrm{Appearance}}_{\mathrm{Plain}}}$, $\beta_{{\mathrm{Form}}_{\mathrm{Chunk}}}$, $\beta_{{\mathrm{Form}}_{\mathrm{Fillet}}}$, and $\beta_{{\mathrm{Color}}_{\mathrm{Red}}}$, for example, remain directionally consistent (i.e., their signs remain unchanged in Table 4), indicating that these effects are robust to prior choice, whereas others like $\beta_{0}$ are much more inconsistent. For instance, the posterior odds for plain samples consistently exceed 1, with 95% credible intervals excluding unity under all priors, providing strong evidence that unmodified appearance may be associated with elevated mislabeling risk. Conversely, red‐colored samples exhibit posterior odds well below 1, with tightly bounded credible intervals, suggesting a consistent protective effect against mislabeling. Estimates for many parameters (e.g., $\beta_{0}$) are consistent yet highly uncertain across priors, given large posterior SDs and wide CrIs. Despite this, CIs and CrIs overlap considerably across priors, yet are also quite wide. This indicates that the seafood mislabeling problem is not as simple as it appears to be on the outset for Bayesian computation to solve. Such a finding strongly suggests that prior elicitation from a domain‐specific expert is likely warranted and would be beneficial, as no clear prior seems to outperform any other across all estimated model parameters for the data at hand. A path forward could be to employ priors specified by Luque and Donlan (2019). Additionally, it stands to reason that expanding the model to the pooled, unpooled, and hierarchical cases, as well as those incorporating spatiotemporal structure, each with fixed/varying intercepts and fixed/varying regression coefficients (slopes), would likely lead to better agreement across selected priors, as well as more realistic interpretation.

**TABLE 4:** Bayesian GLM estimates. Interpretation is identical to that in Table 2.

**Parameter**	**Flat**	**Standard deviation**	**95% credible interval**	**Normal**	**Standard deviation**	**95% credible interval**	**Default**	**Standard deviation**	**95% credible interval**	**Cauchy**	**Standard deviation**	**95% credible interval**
$\beta_{0}$	2.271	1.952	(−1.810, 5.907)	−0.354	0.811	(−1.970, 1.213)	1.879	1.788	(−1.868, 5.225)	0.529	1.382	(−2.200, 3.286)
$\beta_{{\mathrm{Source}}_{\mathrm{Restaurant}}}$	0.964	0.933	(−0.908, 2.814)	0.526	0.579	(−0.573, 1.684)	0.901	0.911	(−0.879, 2.703)	0.766	0.776	(−0.755, 2.290)
$\beta_{{\mathrm{Source}}_{\mathrm{SushiBar}}}$	0.083	0.384	(−0.656, 0.840)	−0.080	0.345	(−0.752, 0.600)	0.060	0.396	(−0.696, 0.841)	−0.003	0.382	(−0.748, 0.728)
$\beta_{{\mathrm{State}}_{\mathrm{Raw}}}$	−1.464	1.076	(−3.753, 0.403)	−0.577	0.579	(−1.726, 0.556)	−1.287	0.989	(−3.336, 0.497)	−0.937	0.825	(−2.662, 0.610)
$\beta_{{\mathrm{Appearance}}_{\mathrm{Plain}}}$	2.171	0.920	(0.597, 4.240)	0.993	0.492	(0.087, 1.986)	1.952	0.831	(0.536, 3.790)	1.513	0.694	(0.285, 3.036)
$\beta_{{\mathrm{Form}}_{\mathrm{Chunk}}}$	−3.816	1.924	(−7.493, 0.193)	−0.766	0.702	(−2.198, 0.628)	−3.386	1.758	(−6.703, 0.329)	−1.915	1.368	(−4.739, 0.707)
$\beta_{{\mathrm{Form}}_{\mathrm{Fillet}}}$	−3.667	1.850	(−7.101, 0.334)	−0.695	0.663	(−1.961, 0.623)	−3.220	1.697	(−6.423, 0.451)	−1.774	1.299	(−4.333, 0.776)
$\beta_{{\mathrm{Form}}_{\mathrm{Whole}}}$	−1.433	2.079	(−5.236, 3.034)	0.731	0.748	(−0.733, 2.208)	−1.021	1.915	(−4.630, 3.020)	0.297	1.423	(−2.383, 3.193)
$\beta_{{\mathrm{Color}}_{\mathrm{Red}}}$	−1.837	0.423	(−2.718, −1.066)	−1.479	0.352	(−2.196, −0.793)	−1.809	0.426	(−2.696, −1.025)	−1.692	0.399	(−2.505, −0.976)

**TABLE 5:** Bayesian GLM odds and probabilities. Interpretation is identical to that in Table 3.

**Parameter**	**Flat**	**95% credible interval**	**Normal**	**95% credible interval**	**Default**	**95% credible interval**	**Cauchy**	**95% credible interval**
${\text{Odds}}_{\text{intercept}}$	9.692	(0.164, 367.652)	0.702	(0.139, 3.364)	6.548	(0.154, 185.848)	1.698	(0.111, 26.746)
${\text{Odds}}_{{\mathrm{Source}}_{\mathrm{Restaurant}}}$	2.622	(0.403, 16.682)	1.693	(0.564, 5.385)	2.463	(0.415, 14.924)	2.152	(0.470, 9.874)
${\text{Odds}}_{{\mathrm{Source}}_{\mathrm{SushiBar}}}$	1.086	(0.519, 2.316)	0.923	(0.471, 1.823)	1.062	(0.499, 2.319)	0.997	(0.473, 2.072)
${\text{Odds}}_{{\mathrm{State}}_{\mathrm{Raw}}}$	0.231	(0.023, 1.496)	0.562	(0.178, 1.743)	0.276	(0.036, 1.644)	0.392	(0.070, 1.841)
${\text{Odds}}_{{\mathrm{Appearance}}_{\mathrm{Plain}}}$	8.765	(1.817, 69.424)	2.698	(1.091, 7.284)	7.046	(1.709, 44.242)	4.542	(1.329, 20.830)
${\text{Odds}}_{{\mathrm{Form}}_{\mathrm{Chunk}}}$	0.022	(0.001, 1.213)	0.465	(0.111, 1.873)	0.034	(0.001, 1.390)	0.147	(0.009, 2.028)
${\text{Odds}}_{{\mathrm{Form}}_{\mathrm{Fillet}}}$	0.026	(0.001, 1.397)	0.499	(0.141 1.864)	0.040	(0.002, 1.569)	0.170	(0.013, 2.172)
${\text{Odds}}_{{\mathrm{Form}}_{\mathrm{Whole}}}$	0.239	(0.005, 20.774)	2.078	(0.481, 9.098)	0.360	(0.010, 20.481)	1.346	(0.092, 24.354)
${\text{Odds}}_{{\mathrm{Color}}_{\mathrm{Red}}}$	0.159	(0.066, 0.344)	0.228	(0.111, 0.452)	0.164	(0.068, 0.359)	0.184	(0.082, 0.377)
${\text{Prob}}_{\text{intercept}}$	0.906	(0.141, 0.997)	0.412	(0.122, 0.771)	0.868	(0.133, 0.995)	0.629	(0.100, 0.964)
${\text{Prob}}_{{\mathrm{Source}}_{\mathrm{Restaurant}}}$	0.724	(0.287, 0.943)	0.629	(0.361, 0.843)	0.711	(0.293, 0.937)	0.683	(0.320, 0.908)
${\text{Prob}}_{{\mathrm{Source}}_{\mathrm{SushiBar}}}$	0.521	(0.342, 0.698)	0.480	(0.320, 0.646)	0.515	(0.333, 0.699)	0.499	(0.321, 0.674)
${\text{Prob}}_{{\mathrm{State}}_{\mathrm{Raw}}}$	0.188	(0.022, 0.599)	0.360	(0.151, 0.635)	0.216	(0.035, 0.622)	0.282	(0.065, 0.648)
${\text{Prob}}_{{\mathrm{Appearance}}_{\mathrm{Plain}}}$	0.898	(0.645, 0.986)	0.730	(0.522, 0.879)	0.876	(0.631, 0.978)	0.820	(0.571, 0.954)
${\text{Prob}}_{{\mathrm{Form}}_{\mathrm{Chunk}}}$	0.022	(0.001 0.548)	0.317	(0.100, 0.652)	0.033	(0.001, 0.582)	0.128	(0.009, 0.670)
${\text{Prob}}_{{\mathrm{Form}}_{\mathrm{Fillet}}}$	0.025	(0.001, 0.583)	0.333	(0.124, 0.651)	0.038	(0.002, 0.611)	0.145	(0.013, 0.685)
${\text{Prob}}_{{\mathrm{Form}}_{\mathrm{Whole}}}$	0.193	(0.005 0.954)	0.675	(0.325, 0.901)	0.265	(0.010 0.953)	0.574	(0.084, 0.961)
${\text{Prob}}_{{\mathrm{Color}}_{\mathrm{Red}}}$	0.137	(0.062 0.256)	0.186	(0.100, 0.311)	0.141	(0.064 0.264)	0.155	(0.076, 0.274)

#### 2.2.3 Prediction and Classification

Estimated mislabeling rates also vary substantially for the four missing samples (Tables 6 and 7). The log odds and odds have the same interpretation in terms of percent changes given previously. For instance, in the case of the frequentist GLM, sample R2 (a cooked modified red‐colored fillet collected from a restaurant) may be associated with an 88.593% lower odds of being mislabeled compared to a cooked modified chopped light‐colored sample from a grocery store.

**TABLE 6:** Predicted frequentist GLM mislabeling log odds, odds, probabilities, and predictions/classifications for missing samples. Labeling status was inferred based on a decision cutoff of 50%. Interpretation of other parameters is identical to that in Tables 2 and 3.

**Sample**	**log odds**	**Odds**	**Probability**	**Outcome**
G80	−2.511	0.081	0.075	Correctly labeled
R2	−2.171	0.114	0.102	Correctly labeled
R51	−0.431	0.650	0.394	Correctly labeled
S10	−0.605	0.546	0.353	Correctly labeled

**TABLE 7:** Predicted Bayesian GLM mislabeling log odds, odds, probabilities, and predictions/classifications for missing samples. Labeling status was inferred based on a decision cutoff of 50%. Calculated values are posterior means averaged over 4000 draws. Interpretation of other parameters is identical to that in Table 6.

**Prior**	**Sample**	**log odds**	**Odds**	**Probability**	**Outcome**
Flat	G80	−2.442	0.087	0.080	Correctly labeled
Flat	R2	−2.122	0.120	0.107	Correctly labeled
Flat	R51	−0.343	0.710	0.415	Correctly labeled
Flat	S10	−0.541	0.582	0.368	Correctly labeled
Normal	G80	−1.537	0.215	0.177	Correctly labeled
Normal	R2	−1.883	0.152	0.132	Correctly labeled
Normal	R51	−0.485	0.616	0.381	Correctly labeled
Normal	S10	−0.654	0.520	0.342	Correctly labeled
Default	G80	−2.351	0.095	0.087	Correctly labeled
Default	R2	−2.111	0.121	0.108	Correctly labeled
Default	R51	−0.410	0.664	0.399	Correctly labeled
Default	S10	−0.549	0.578	0.366	Correctly labeled
Cauchy	G80	−2.021	0.133	0.117	Correctly labeled
Cauchy	R2	−2.031	0.131	0.116	Correctly labeled
Cauchy	R51	−0.435	0.647	0.393	Correctly labeled
Cauchy	S10	−0.619	0.538	0.350	Correctly labeled

Overall, the model predicts all missing samples are likely to be correctly labeled under the default decision boundary for both the frequentist and Bayesian GLMs, as all estimated probabilities are below 50%. Despite this, species identification, along with other sample properties, could not be made, as mentioned in Section 3.1. Nevertheless, prediction of samples with unknown labeling status is useful to verify whether findings agree well with observed food processing and fraudulent behaviors, such as removal of morphological features that readily distinguish among species (e.g., skin, fins, and scales), concealing at‐risk seafood species within highly preserved canned goods, and adding dyes or pigments to farmed Atlantic salmon (*Salmo salar*) to pass it off as wild‐caught Pacific (Sockeye) salmon (*Oncorhynchus nerka*).

As with estimates for model parameters, results depicted in Tables 6 and 7 demonstrate that the detection of mislabeled seafood is challenging under the chosen priors and default classification threshold, as all estimates are very close in magnitude. Given the strong degree of class imbalance toward correctly labeled seafood samples collected by Hu et al. (2018) (especially the Form category), it does not seem surprising that the logistic model employed in the current work classified all four missing‐label status samples as not likely mislabeled. Thus, future work should aim to investigate robust balancing techniques such as upweighting and downsampling of minority and majority classes, respectively, to minimize bias as much as possible.

## 3 Discussion

### 3.1 Summary

In the present study, established but rigorous statistical theory was employed as a teaching tool to educate those, particularly in regulatory fields, on the impacts of fraudulent seafood labeling practices in a holistic fashion, from policy to behavior. Herein, a case study on seafood mislabeling trends in Metro Vancouver, British Columbia, Canada by Hu et al. (2018) was utilized to demonstrate the overall promise of frequentist and Bayesian statistical modelling to estimate seafood mislabeling rates, as well as highlight implications for legislation, policy, and health impacts around the import and sale of both common and at‐risk species on a global scale. Hu et al. (2018) employed a statistical approach that may benefit from further clarification or refinement. Given that the article has been cited over 120 times as of April 2026, it appears the findings have been widely referenced, though perhaps not extensively reexamined in terms of their underlying analysis. It needs to be stressed that results obtained here for the frequentist analysis differ markedly from those of Hu et al. (2018). Further, findings from the Bayesian analysis aptly demonstrate that application of the approach to estimate seafood mislabeling rates within the supply chain is not so clearcut. Practitioners must recognize the limitations of data, models, analyses, and even software when it comes to answering fundamental questions concerning aspects of seafood fraud.

Here, it was found that seafood samples obtained from restaurants and sushi bars have a higher chance of being mislabeled compared to those found in grocery stores, which agrees well with current practices, especially in the United States (e.g., Ahles et al. 2025; Garcia et al. 2024; Kitch et al. 2023). Additionally, seafood samples having a plain appearance were over six times more likely to be incorrectly labeled compared to modified samples. Such findings are in line with global trends. For example, product mislabeling rates are often higher in restaurants in comparison to grocery stores because restaurants tend to purchase large volumes of seafood from several different suppliers, distributors, or wholesalers, in sharp contrast to grocery stores who acquire their seafood supply from more homogeneous sources. This in turn leads to problems in transparency, as there are few regulations and limited enforcement specifically targeting restaurants. Although large statistical effects for many model covariates were found herein, it is important to remember that they are only *suggestive*, especially for those seafood product subcategories with low observed sample sizes like chopped, chunk, and whole form, as well as those subcategories which show strong class imbalance.

The primary objective of this study was estimation of overall patterns in seafood mislabeling rather than inference regarding specific supply chain effects. While unmodelled clustering may influence uncertainty estimates, it is less likely to substantially alter the estimated direction or magnitude of broad population‐level trends. Potential clustering among products from common supply chains could induce residual dependence among observations. Such dependence would primarily affect precision estimates rather than point estimates themselves and therefore should be considered when interpreting uncertainty intervals. Hierarchical modelling also has the ability to better regularize and stabilize parameter estimates, leading to narrower confidence and credible intervals not seen in the current study. Within Hu et al. (2018), the majority of seafood products were collected from Vancouver, including all samples from sushi establishments, suggesting a strong spatial effect. Future studies designed with explicit geographic and supply chain metadata in mind would permit implementation of hierarchical approaches and more rigorous evaluation of dependence structures to enable estimation of variance components. Such information could be gleaned from Table 2 of Hu et al. (2018). However, note there is considerable mismatch between sample IDs when compared to the authors' GLM data (Supporting Information): In Hu et al.'s (2018) paper, seafood samples are listed out of order (i.e., 12, 20, 25, …), whereas their provided dataset enumerates data in numeric order (i.e., 1, 2, 3, …). It is unclear whether there is exact correspondence between these two data sources. Matching these samples exactly would not be straightforward; hence, this is further reason why a fixed‐effects model, rather than a hierarchical one, was adopted for the current study. This paper highlights that statistical approaches are important because accurate estimation of seafood mislabeling prevalence can directly inform fraud surveillance, regulatory oversight, and authentication strategies. Beyond methodological contributions, the present framework outlined here may provide practical value for seafood authentication programs, risk‐based inspection efforts, and development of traceability systems aimed at reducing seafood fraud. More advanced statistical inference, prediction, and classification methods which should be explored in frequentist and Bayesian contexts are given in the Supporting Information.

### 3.2 Past Studies

The application of statistical models, such as GLMs, to address standing questions in seafood fraud, is not new; however, much of the existing research has either relied on relatively narrow analytical approaches or adopted more complex methodologies while advancing broad conclusions. Some studies, including that of Hu et al. (2018), also present challenges in terms of data/code availability and reproducibility, slowing uptake within the broader food science sector.

A controversial study by Stawitz et al. (2016) that examined both the financial impact and the ecological ramifications of global seafood product fraud for a variety of fish species (6754 samples spanning 43 DNA barcoding publications) employed a frequentist GLM framework in combination with model selection using the Akaike information criterion (AIC) (Akaike 1974) given by

(4) $$\text{AIC}=2k-2\log\hat{L}.$$

where k is the number of parameters and $\hat{L}$ is the MLE. The authors utilized taxonomic information, in addition to species price data and species conservation status to develop logistic regression models in an effort to determine the overall effects of seafood mislabeling for samples collected from six sources: port, distributor, grocery, market, restaurant, and sushi (Stawitz et al. 2016). However, there were several major limitations to this work. The best GLM in terms of lowest AIC reported by Stawitz et al. (2016), which incorporated genus, country, and purchase source information as covariates for the five most commonly consumed finfish groups in the United States (Atlantic and Pacific salmon, tuna, catfish, and cod), found that species tested using DNA barcoding were both cheaper (2.98% ex‐vessel price) and of higher conservation status (+3.88% IUCN conservation status) compared to the species names listed on product labels. First, the use of the IUCN Red List status was very crude. For products that could be identified to the genus or species level, which accounted for 32.520% of samples, the authors used the average status of the respective species, which is entirely inaccurate, due to varying conservation status at the genus level. This led the researchers to conclude that a label can often be of more severe conservation status than the true species, which was contradictory to most other research. The authors argued that if species of lower conservation priority are being used to substitute species of higher conservation concern, then product mislabeling might actually be increasing seafood sustainability. From an interpretive perspective, in reality, this is far from the truth. However, this conclusion may be an oversimplification. Within the seafood supply chain, the opposite trend is also seen. In some cases, higher valued species are deliberately mislabeled as lower valued ones, for example, to obscure their origin from IUU fishing activities (Gordoa et al. 2017; Lorusso et al. 2024). Stawitz et al.'s (2016) findings and overall conclusions were immediately called into question in a number of rebuttals (Donlan et al. 2017; Mariani et al. 2017; Warner et al. 2017). Criticism revolved around the application of the USFDA Seafood List to inform global species mislabeling trends and the use of inappropriate GLM covariates to estimate mislabeling rates, among other misapplications. In response to unfavorable attention generated from Stawitz et al. (2016), especially by Donlan et al. (2017), who noted “seafood fraud deserves better,” Siple et al. (2017) admit clear statistical oversights.

Other studies have employed statistical regression methods in an effort to better assess global seafood mislabeling impacts at many hierarchical levels, but most are either inconsistent in methodology, or incomplete in analysis. Most studies simply employ basic one‐way or two‐way tests of independence or goodness of fit, but several studies have implemented more advanced methods (e.g., Kim and Lee 2018; Munguia‐Vega et al. 2022; Stawitz et al. 2016; Luque and Donlan 2019). Kim and Lee (2018) utilized ordered probit regression to model consumer purchasing and consumption preference to eco‐labeled seafood (flatfish, salmon, tuna, and octopus) across 2773 households in Korea, accounting for covariates such as seafood freshness and price, as well as age, sex, and income of survey respondents. Munguia‐Vega et al. (2022) used log‐transformed linear models to investigate the global and local influences on patterns of mislabeling and substitution in the Mexican fish trade sector. Perhaps to amend Stawitz et al.'s (2016) misunderstandings, Luque and Donlan (2019) conducted a large‐scale Bayesian meta‐analysis of global seafood fraud studies totaling 27,313 samples across 141 usable papers (both peer‐reviewed and unrefereed) at seven levels including country, source (restaurant, port, market, and grocery), year, product form (processed and fillet), and taxonomic identity (genus and species). Using a two‐stage hierarchical modelling framework, an overall product‐level mislabeling rate of 8% (95% HPDI [Highest Posterior Density Interval]: 4%–14%) was noted.

### 3.3 How Can a Statistical Framework of Seafood Product Fraud Help Moving Forward?

The statistical modelling framework of seafood fraud introduced here is a general one that can be employed in several ways. Specifically, it can be utilized to fulfill numerous short‐term and long‐term goals of importance to interested parties in several ways, including, but not limited to:

- Capturing geographic variability of seafood species fraud (e.g., adulteration, mislabeling, substitution etc.) within the supply chain for metropolitan cities across Canada and the United States, as well as coastal areas of Europe and Asia for the most problematic species, which include cod, halibut, salmon, sole, snapper, and tuna.
- Reviewing and evaluating existing trends in seafood product fraud for widely consumed species in terms of biomass availability, market price, and conservation status.
- Employing and adopting developed models in academic, regulatory, and collaborator settings to project seafood fraud time of occurrence within major and minor Canadian and US cities for widely misrepresented species at different levels of the supply chain.
- Developing improved supply chain traceability detection measures and control strategies/guidelines leading to lower prevalence and incidence of seafood fraud in the Canadian and American markets and elsewhere.
- Implementing robust policy and legislation to combat seafood fraud based on statistically defensible model predictions of mislabeling rates and time of occurrence for species of economic, environmental, and health concern.

Taken together, these objectives will better aid decision‐making by both regulators and consumers. Consumers need to be acutely aware of what they are purchasing and know the telltale signs (both good and bad) to look for, as well as the right questions to ask. Who caught and processed the samples? Where? When? How? Buying eco‐labeled products with stamps of approval from non‐governmental organizations (NGOs) like SeaChoice, OceanWise, or the Marine Stewardship Council (MSC) is definitely a step in a positive direction, but even brands bearing these trustworthy seals are not immune to the clutches of mislabeling. A new report released in February 2026 by the Food and Agriculture Organization (FAO) found that up to 20% of seafood is affected by fraud globally, with rates in excess of 75% in some cases (Food and Agriculture Organization of the United Nations 2026). Furthermore, regulatory agencies in both Canada and the United States should work to adopt better seafood labeling conventions to mitigate fraud by more closely emulating European neighbors. This includes the use of scientific names rather than, or in addition to, common species names on product packaging, indication of products' production method (e.g., wild‐caught vs. farm‐raised), as well as upholding Country of Origin Labeling (COOL), that is, specifying the location where seafood was caught as opposed to where it was processed, a practice that is already implemented within Canada by the CFIA and the United States through the USFDA (Naaum et al. 2015; Silva et al. 2021). Inclusion of common species names in other languages like Japanese for well‐known species employed in dishes like sushi may also help reduce levels of seafood fraud (Willette et al. 2025). Maintaining existing partnerships and establishing new alliances between nations is crucial to effectively combating fraud through improving seafood product traceability and implementing advanced detection methods. The Canada‐European Union Comprehensive Economic and Trade Agreement (CETA) is one such link that could aid in this endeavor (Shehata, Naaum, et al. 2019). While seafood mislabeling and other types of product fraud have been on steadily the rise despite intervention efforts, a 10‐year longitudinal study carried out in Los Angeles from 2012 to 2023 saw a threefold *decrease* in fraudulent labeling practices from 46.835% between 2012 and 2015 to 34.006% from 2018 to 2023 due in large part to a combination of outreach and DNA barcoding initiatives by the Los Angeles Seafood Monitoring Project, a voluntary engagement program involving seafood restaurants, local universities, and government agencies aiming to reduce mislabeling rates using best practices and evidence‐based recommendations (Willette et al. 2017).

Although the present study primarily focused on statistical estimation and uncertainty quantification, the findings also have broader implications for seafood authentication and fraud prevention efforts. Reliable estimation of seafood mislabeling prevalence can help identify contexts in which substitution risk may be elevated and where monitoring resources may be most effectively allocated. From a food authentication perspective, DNA barcoding and related molecular approaches provide powerful tools for identifying substitution events throughout seafood supply chains. However, effective authentication requires not only analytical tools but also statistically robust sampling frameworks capable of generating reliable prevalence estimates. Improved sampling design may help regulatory agencies distinguish localized substitution events from broader systemic patterns. The present findings also have potential implications for regulatory decision‐making. Agencies responsible for food inspection and consumer protection frequently operate under limited resources, making risk‐based prioritization important. Quantitative estimates of uncertainty may assist in identifying product categories, taxa, or market sectors warranting increased surveillance. Traceability systems may further strengthen seafood fraud prevention by improving transparency across supply chains. Digital traceability frameworks, standardized product documentation, and chain‐of‐custody systems could facilitate identification of points where substitution risk is greatest. Integration of molecular authentication approaches with supply chain metadata may provide opportunities for more targeted monitoring strategies. From an industry perspective, improved estimates of mislabeling prevalence may support quality assurance initiatives and strengthen consumer confidence. Producers and retailers implementing robust authentication and traceability measures may reduce reputational risk while improving supply chain accountability.

## 4 Conclusion

Taken together, findings of this study indicate that seafood product characteristics may influence observed patterns of mislabeling risk, although substantial uncertainty remains due to small sample sizes and extreme class imbalance. Obtained model estimates provide insight into broad trends of seafood fraud rather than definitive evidence of causal mechanisms. Nevertheless, results are insightful as they provide a potential mechanism as to *how* seafood fraud, such as mislabeling, is occurring within the supply chain through alteration of physical properties of collected products, as well as *where* mislabeling is most prevalent. To address the *when* aspect, sophisticated time series models are likely needed together with spatial approaches. However, it is important not to make over‐generalizations concerning findings described within the current study: results should only be interpreted in the context of seafood fraud incidence and prevalence within Metro Vancouver. Nevertheless, the integration of frequentist and Bayesian statistical methods into future studies holds much promise.

## Author Contributions

J.D.P. wrote the manuscript, wrote R and Stan code, approved all developed code as well as analyzed and interpreted all experimental results. F.A.D.V.‐F wrote required R and/or Stan code. All authors contributed to the revision of this manuscript and approved the final version.

## Funding

This research was undertaken thanks in part to funding from the Canada First Research Excellence Fund (CFREF‐2015‐00004).

## Conflicts of Interest

The authors declare no conflicts of interest.

## Supporting information



Data S1

## Data Availability

Raw data, R, and Stan code can be found on GitHub at https://github.com/jphill01/Phillips‐et‐al.‐Seafood‐Fraud‐Paper.
