# Supplementary material for: Statistical Modeling of Seafood Fraud Highlights Uncertainties in Products From Metro Vancouver, British Columbia, Canada: Revisiting Hu et al. (2018)
Source: J Food Sci. 2026 Jun 18;91(6):e71201. doi: 10.1111/1750-3841.71201 (PMC13279869; doi:10.1111/1750-3841.71201)
Supplement: Supplementary file 1 — Data S1 [file JFDS-91-0-s001.pdf]

Statistical modelling of seafood fraud highlights uncertainties in products from Metro Vancouver, British Columbia, Canada: Revisiting Hu *et al.* (2018)

Supplementary Information

Jarrett D. Phillips and Fynn A. De Vuono-Fraser

Data Summary

Factor level	Correctly labelled	Mislabelled
Grocery (G)	70	22
Restaurant (R)	58	24
SushiBar (S)	83	24

Table 1: Correctly labelled and mislabelled samples by source.

Factor level	Correctly labelled	Mislabelled
Cooked	62	25
Raw	149	45

Table 2: Correctly labelled and mislabelled samples by state.

Factor level	Correctly labelled	Mislabelled
Modified	96	28
Plain	115	42

Table 3: Correctly labelled and mislabelled samples by appearance.

Factor level	Correctly labelled	Mislabelled
Chopped	2	1
Chunk	24	3
Fillet	183	61
Whole	2	5

Table 4: Correctly labelled and mislabelled samples by form.

Factor level	Correctly labelled	Mislabelled
Light	116	61
Red	95	9

Table 5: Correctly labelled and mislabelled samples by colour.

Frequentist Analysis

The potential presence of multicollinearity among predictors was assessed via the `vif()` function in the `car` package (Fox and Weisberg, 2019) using default argument settings. `vif()` computes Variance Inflation Factors (VIFs) based on regressing each predictor onto all others and then reporting the coefficient of determination (R^2). The VIF is given by:

$$VIF = \frac{1}{1 - R_i^2}, \quad (1)$$

where variables with VIF > 5 (equivalent to an $R^2 > 0.800$ from rearranging Equation (5)) should be dropped from the model since their inclusion could lead to unstable behaviour of outputted estimates and interpretation could become challenging due to lack of independence. For instance, in a given logistic regression model, the log odds for a particular variable would no longer necessarily increase by β_i units when said variable is increased by a single unit. When regression models contain many predictors, there is a strong chance that a number of covariates are highly or perfectly correlated (having correlation 1 or -1) with one another. This can lead to identifiability issues since one predictor is a linear function of the other. While some main effects were found to not be significant at the 5% level (**Table 2**), such predictors were not removed from the model due to their perceived importance in the seafood fraud detection literature and the desire to replicate past findings and to mitigate omitted variable bias. This said, it would be worthwhile as part of future work to employ model selection procedures, such as stepwise regression using criteria like the AIC, to arrive at a simpler and more explanatory model. Multicollinearity was dealt with through calculation of the generalised variance inflation factor (GVIF) which is an extension of the VIF for models containing categorical variables. Here, a scaled version of the GVIF, sGVIF, is employed, which is given by:

$$sGVIF = VIF^{\frac{1}{2df}} \quad (2)$$

where *df* is the number of coefficients in a subset of the full model. Given sGVIFs were all less than five, each predictor was kept in the model (**Table 6**). Despite this, sGVIFs for

both Appearance and Form were larger than those for the remaining covariates, suggesting that perhaps one of these predictors is problematic and therefore should be excluded. This is left for a followup study.

**Variable**	**GVIF**	**df**	**sGVIF**
Source	8.817	2	1.723
State	10.290	1	3.208
Appearance	6.986	1	2.643
Form	1.961	3	1.119
Colour	1.094	1	1.046

Table 6: Predictor sGVIFs and other related quantities. All predictors have sGVIF < 5, indicating little to no evidence of multicollinearity.

Bayesian Analysis

Theoretical Background and Computational Details

Unlike frequentist inference, its Bayesian counterpart treats unknown parameters (β) as random variables proportionally through Bayes' theorem to derive a joint posterior distribution from the likelihood ($p(y|X, \beta)$) and an *a priori* chosen prior distribution ($p(\beta)$):

$$p(\beta|X, y) \propto p(y|X, \beta)p(\beta) \tag{3}$$

(Gelman et al., 2014; van de Schoot et al., 2021), where equality holds through division of the right-hand side of the above expression by the prior predictive distribution $p(y|x) = \int p(y|X, \beta)p(\beta)\,\mathrm{d}\beta$, in which model parameters are marginalised out. However, often this integral has no analytical (*i.e.*, closed-form) solution, thus necessitating numerical approximations. Therefore, Hamiltonian Monte Carlo (HMC) was employed to generate dependent samples from the posterior distribution of interest. HMC is inspired by Hamiltonian mechanics, wherein a proposed sample moving toward the target distribution is likened to the movement of a particle in a physical system based on its position, velocity, energy, and momentum over time (Neal, 2011). Compared to other Markov Chain Monte Carlo (MCMC) algorithms like the Metropolis-Hastings sampler (Hastings, 1970) and the Gibbs sampler (Geman and Geman, 1984), HMC sampling in Stan is much faster computationally and is much more efficient for challenging posteriors in terms of chain mixing compared to sister software like JAGS (Just Another Gibbs Sampler) (Plummer, 2003). This is the case since HMC relies on gradient (*i.e.*, multivariate derivative) information to explore the parameter search space, whereas JAGS imposes no such overhead, as it is essentially a random walk. Further, because resulting samples are minimally autocorrelated, Markov chain thinning is not required. Thus, convergence to target densities is quite rapid and acceptance rates of proposed samples is optimal. This behaviour is especially desirable in high-dimensional situations, where the number of model parameters exceeds the number of sampled observations. In the context of seafood fraud, product mislabelling rate estimation

can be viewed as a high-dimensional problem as few samples are often collected across multiple predictors (*e.g.*, source, geographical location, time).

Many past studies employing Bayesian analysis centre prior distributions on MLEs; however, this "double dipping" is bad practice for two reasons (van de Schoot et al., 2021). First, priors should be specified independently of the data (*i.e.*, before seeing any data or a statistical summary thereof). Secondly, centering of prior distributions results in inflation of posterior effective sample sizes (ESSs; see below) relative to actual parameter sample sizes, leading to overconfidence in obtained results. Note, Stan parameterises models using the standard deviation, not the variance. Convergence was assessed in a number of ways as suggested by Gelman et al. (2020): (1) through examining parameter traceplots, which depict the trajectory of accepted MCMC draws as a function of the number of iterations, (2) through monitoring the Gelman-Rubin $\hat{R}$ statistic (Gelman and Rubin, 1992; Vehtari et al., 2021), which measures within-chain *versus* between-chain variance, and (3) through calculating the ESS for each parameter, which quantifies the number of independent samples generated Markov chains are equivalent to. Mixing of chains was deemed sufficient when traceplots looked like "fuzzy caterpillars", $\hat{R} < 1.01$, and effective sample sizes were reasonably large.

All results shown below are based on 4000 posterior draws. Across all examined priors, parameter traceplots showed rapid convergence to the stationary distribution (**Figures 9-12**), and effective sample sizes (not shown) were all above 1000.

Addressing Computational Issues

When extending the present statistical framework to accommodate hierarchical models, a common distribution for hyperparameters and hyperpriors could be easily specified. However, this could result in a lower degree of interpretability due to the adoption of a more complicated model with a larger number of parameters estimated from relatively little data, as well as sampling pathologies commonly encountered in HMC when exploring posterior parameter spaces possessing complex geometries (*e.g.*, curvature). In the latter case, divergent transitions commonly arise, which may prove difficult to diagnose and resolve. A path forward could be to increase the target average acceptance probability (`adapt_delta`), which is set to 0.800 by default in `rstan`, to a value like 0.999. Doing so will result in smaller step sizes taken by the leapfrog integrator, in addition to increasing computational expense. However, the end result will be more robust due to more efficient sampling. Another approach to remedy HMC sampling difficulties, especially in hierarchical settings, is model reparameterisation, in particular centering, which can often achieve convergence in fewer iterations and with larger effective sample sizes (Betancourt and Girolami, 2013); however, implementation can prove challenging.

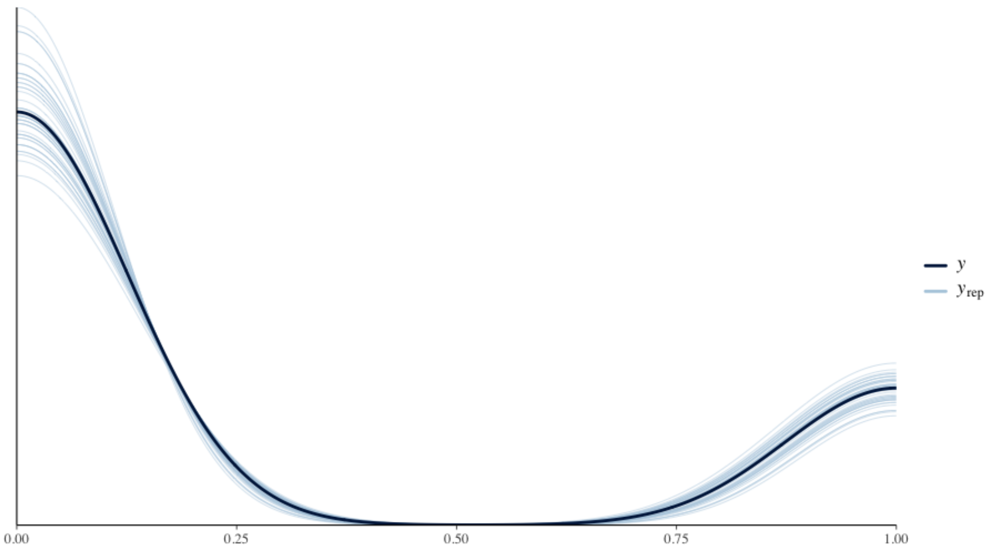

Figure 1: Posterior predictive density for $U(-\infty, \infty)$ & $N(0, 2.5)$ prior distribution.

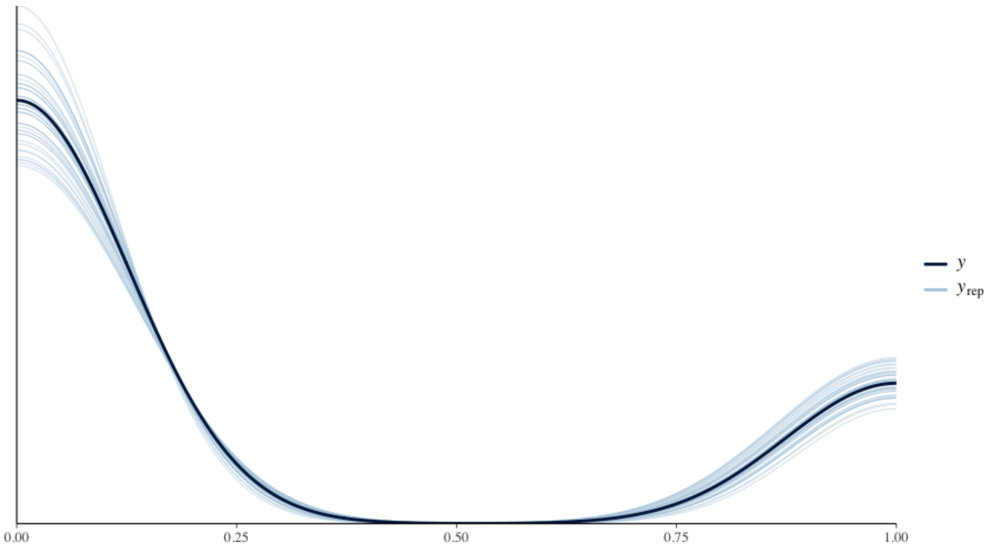

Figure 2: Posterior predictive density for $N(0, 1)$ prior distribution.

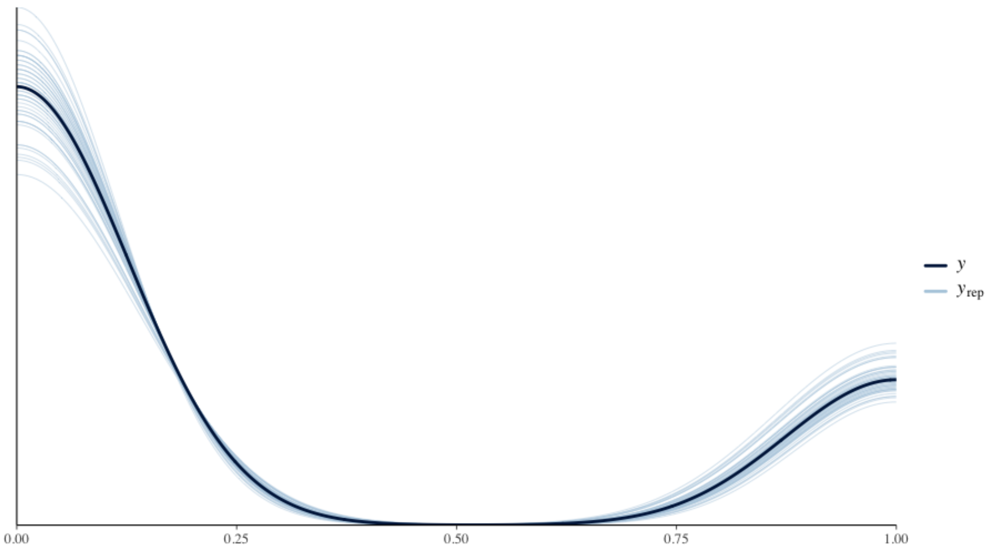

Figure 3: Posterior predictive density for $N(0, 2.5)$ prior distribution.

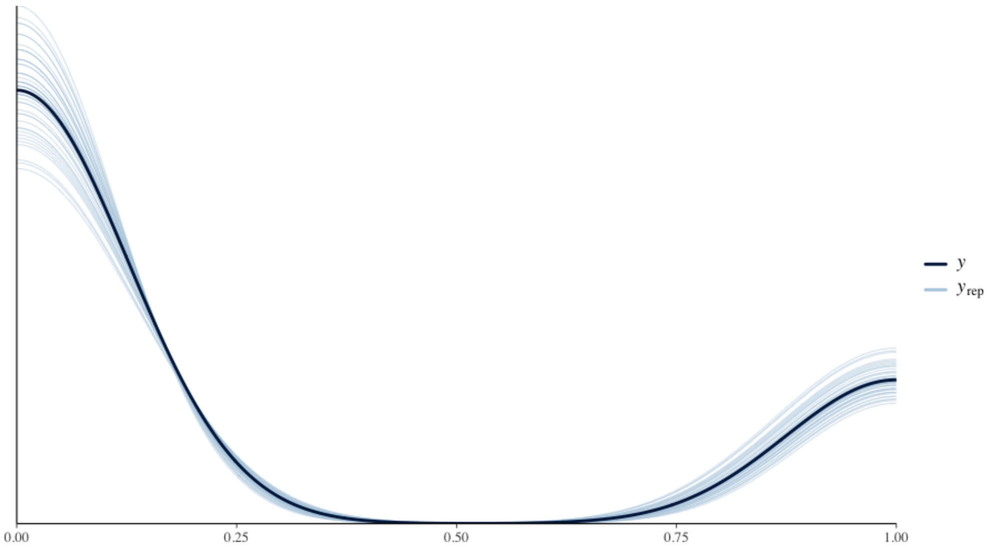

Figure 4: Posterior predictive density for $Cau(0, 10)$ & $Cau(0, 2.5)$ prior distribution.

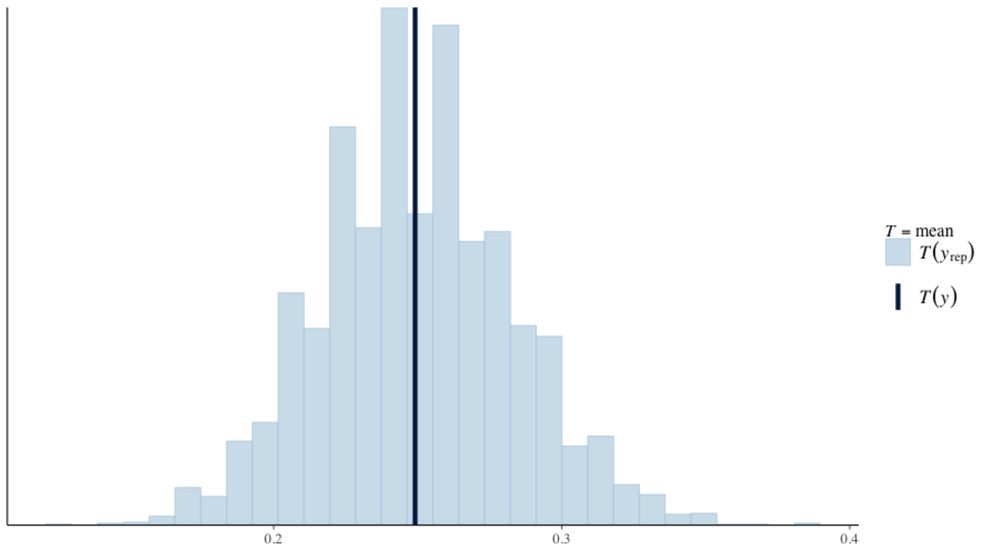

Figure 5: Posterior predictive distribution for $U(-\infty, \infty)$ & $N(0, 2.5)$ prior distribution.

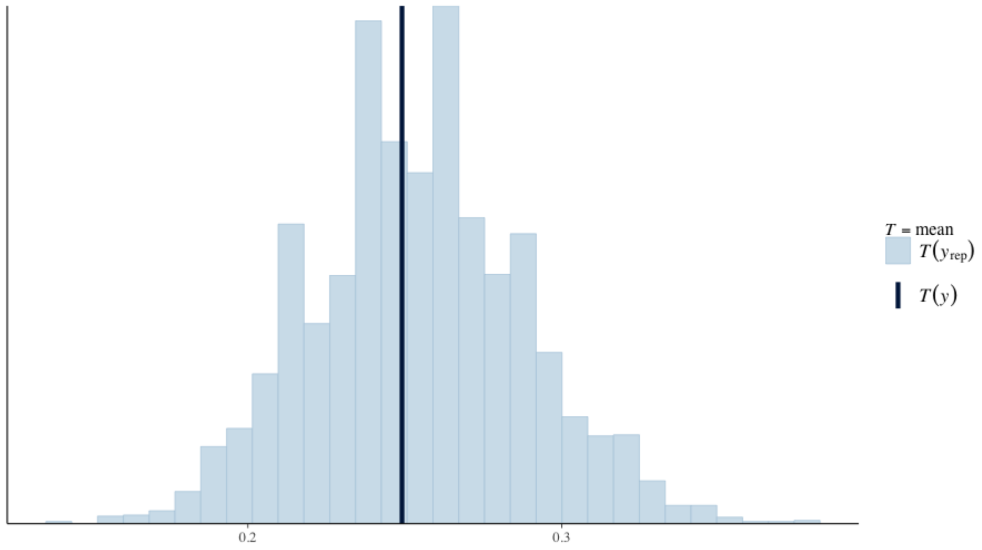

Figure 6: Posterior predictive distribution for $N(0, 1)$ prior distribution.

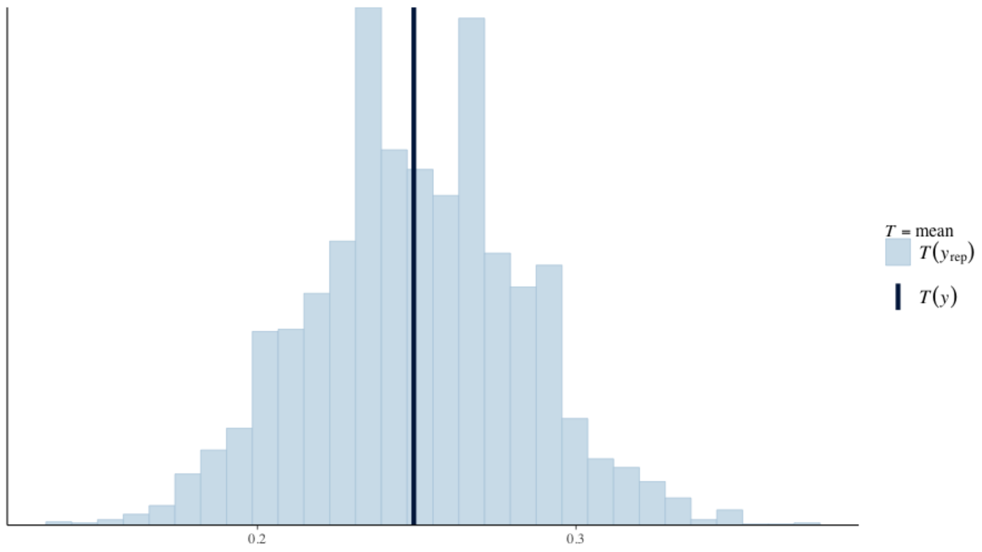

Figure 7: Posterior predictive distribution for $N(0, 2.5)$ prior distribution.

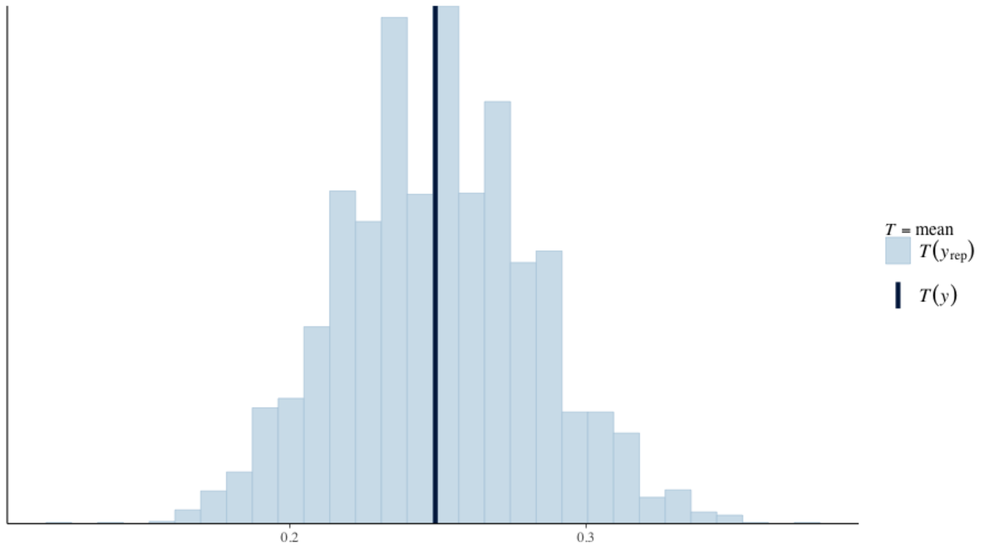

Figure 8: Posterior predictive distribution for $Cau(0, 10)$ & $Cau(0, 2.5)$ prior distribution.

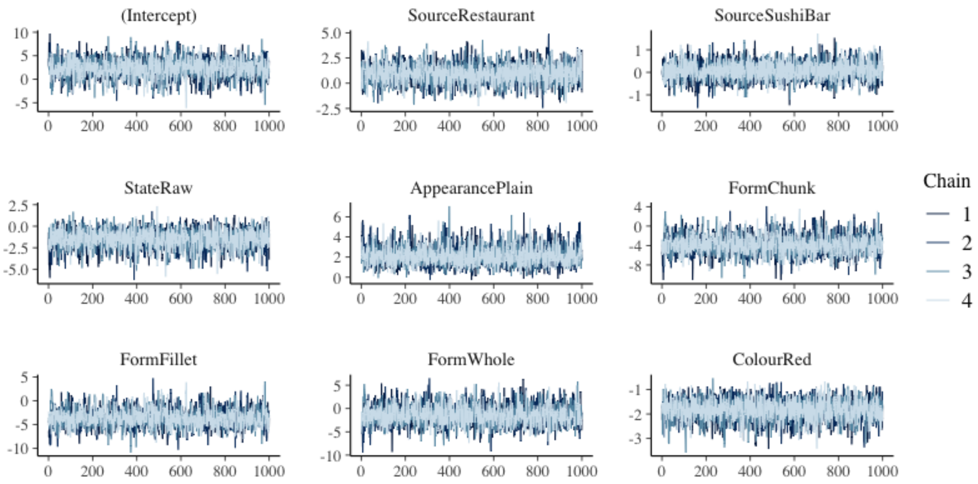

Figure 9: Traceplots for $U(-\infty, \infty)$ & $N(0, 2.5)$ prior distribution on both the intercept and regression coefficients, respectively.

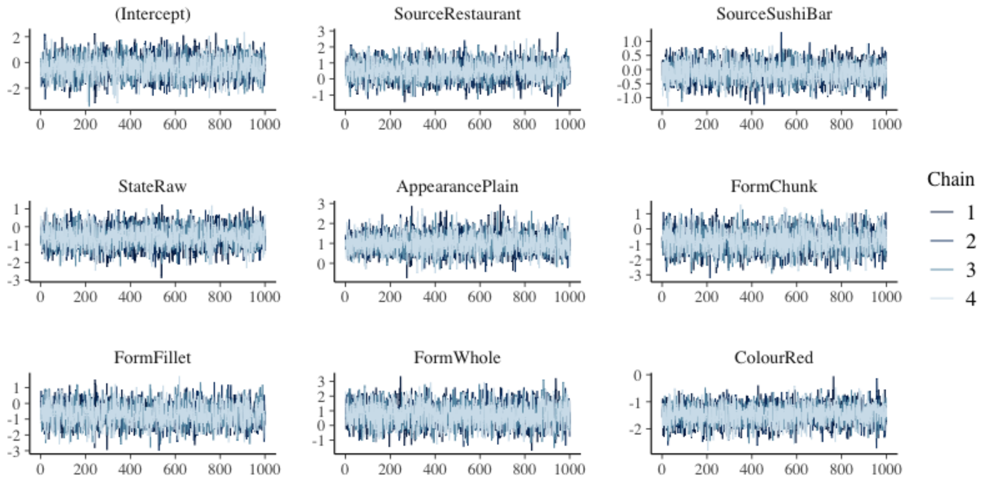

Figure 10: Traceplots for $N(0, 1)$ prior distribution on both the intercept and regression coefficients.

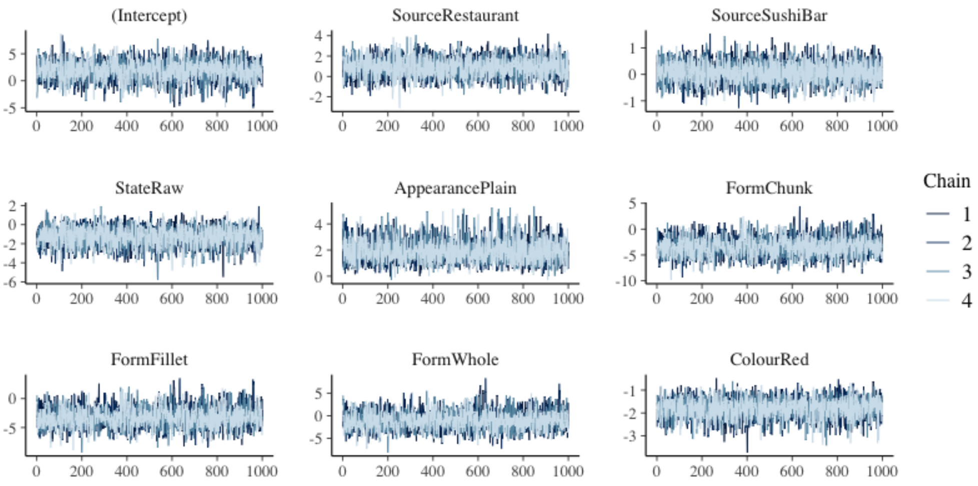

Figure 11: Traceplots for $N(0, 2.5)$ prior distribution on both the intercept and regression coefficients..

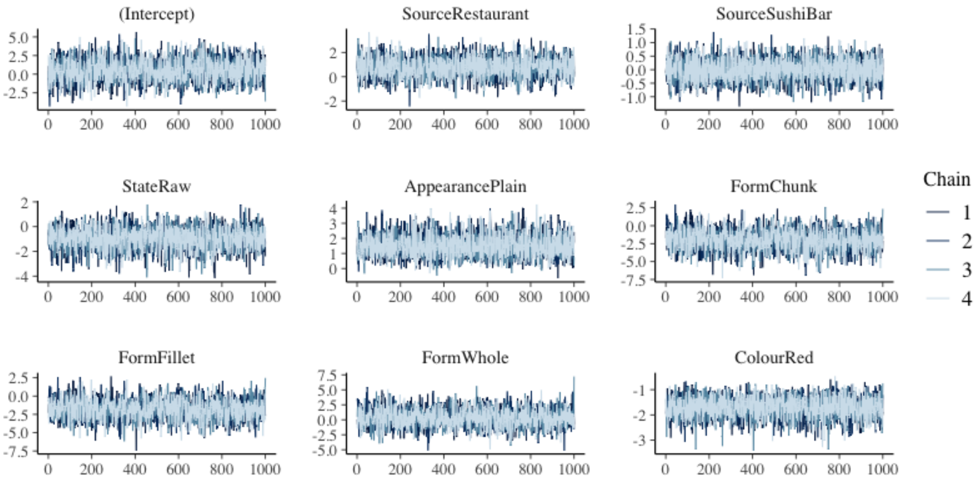

Figure 12: Traceplots for $Cau(0, 10)$ & $Cau(0, 2.5)$ prior distribution on both the intercept and regression coefficients, respectively.

Modelling Extensions

Logistic regression is a widely used modelling approach within many scientific fields. Despite this, it appears not to have been employed before in the context of assessing the impact of seafood fraud through estimation of product mislabelling rates. While this approach was leveraged for the present study, and many model improvements are proposed, a natural extension to probit regression as was similarly carried out by Kim and Lee (2018) is entirely feasible. Logit and probit models could then be compared for goodness of fit via the AIC, the Likelihood Ratio Test (LRT), leave-one-out cross validation in a Bayesian context to compare models fitted using a range of prior distributions via the `loo` R package (Vehtari

et al., 2017, 2023), or techniques like Bayesian model averaging/stacking (Yao et al., 2017) to combine plausible models based on their posterior probabilities. Despite this, probit model interpretation is more challenging and less intuitive compared to logistic models, as coefficients are on the z-score scale and probabilities must be computed numerically via integration. This is the case since the probit is based on the cumulative distribution function (CDF) of the standard Normal distribution. Furthermore, while the two approaches often give comparable output, estimated probabilities may differ significantly, potentially leading to conflicting prediction outcomes regarding product labelling status.

A second consideration concerns calculation of binomial CIs and CrIs themselves. While none of the calculated intervals exhibited problematic behaviour (such as values falling outside their permissible ranges), steps should be taken to ensure this does not occur, especially when sample sizes are small. Another concern is the calculated interval estimates could possess poor nominal coverage; that is, intervals may not reach or exceed 95% confidence. In the case of CIs, methods like the exact Clopper-Pearson interval, Wilson score interval, or Agresti-Coull interval should be used over the standard Wald interval (Newcombe, 1998). Herein, central (equal-tailed) intervals were computed since this is the default in both R and Stan. For bounded quantities like odds and probabilities which have asymmetric distributions, this is not desirable. The use of highest posterior density (HPD) intervals (Chen and Shao, 1999) or shortest probability intervals (SPIns) (Liu et al., 2015) may be worth exploring as alternatives to CrIs for seafood mislabelling rates.

With respect to classification, recall a value of 50% was utilised herein as a decision threshold. Depending on the importance of confidently classifying samples as either correctly labelled or mislabelled (through minimising the number of false positives and false negatives), a different cutoff may be desired. Small thresholds are to be preferred whenever correctly classifying truly mislabelled seafood samples as mislabelled is absolutely critical; however, this comes at a cost of less certainty. Conversely, the use of large cutoffs would result in fewer truly mislabelled samples being detected with greater certainty. To this end, another avenue for future work revolves around classification, where the use of receiver operating characteristic (ROC) curves, which plot the false positive rate (FPR; $1 -$ specificity) against the true positive rate (TPR; sensitivity) and the area bounded by them (area under the curve; AUC), can be utilised on training, validation, and testing sets to select optimal thresholds for seafood sample classification found by maximizing Youden's J statistic, given by

$$J = \text{sensitivity} + \text{specificity} - 1 \tag{4}$$

in conjunction with measures of performance evaluation such as model accuracy, precision, and recall. Youden's J ranges between zero and one, with $J = 0$ suggesting a classification is no better than random chance, and $J = 1$ indicating a perfect classification (*i.e.*, all true positives and all true negatives are correctly identified). Because Hu et al.'s (2018) study is limited in size and scope, any machine learning model generated from it should be viewed with caution. Further, in addition to lack of independence, their dataset exhibits strong class imbalance: 211 correctly labelled samples *versus* 70 mislabelled samples. Therefore, more robust methods like precision-recall curves should also be generated.

Additionally, use of the complementary log-log (cloglog) GLM link function should be explored as it is ideal for rare event data like seafood product mislabelling. Results could

then be compared to logit and probit models to assess model fit. The four seafood samples having unknown labelling status (**Table 1**) were held out from the model and used to assess its predictability. A useful (albeit, perhaps simpler) next step would be to utilise data imputation to classify missing values, using the `mice` R package (van Buuren and Groothuis-Oudshoorn, 2011), for example. Past work by Austin and van Buuren (2023) show this to be a promising alternative; however, predictor variable collinearity is a known issue for imputation. Given that both seafood State and Appearance possess larger than expected variance inflation factors (VIFs) (**Table 6**), this suggests that the use of `mice` may not be so straighforward.

Finally, and perhaps most importantly, is the extension of the fixed-effects logistic regression model to the hierarchical setting. As noted throughout this paper, Hu et al. (2018) left out many important details regarding the source of obtained seafood, among other shortcomings. Inclusion of metadata such as product supplier/distributor within a mixed modelling framework would facilitate a deeper understanding as to the extent of seafood fraud, paving the way for implementation of more robust product authentication strategies, enhanced traceability systems, and development of practical industry applications.

References

Austin, P. C. and S. van Buuren
2023. Logistic regression vs. predictive mean matching for imputing binary covariates. *Statistical Methods in Medical Research*, 32(11):2172–2183.

Betancourt, M. and M. Girolami
2013. Hamiltonian Monte Carlo for hierarchical models.

Chen, M.-H. and Q.-M. Shao
1999. Monte Carlo estimation of Bayesian credible and HPD intervals. *Journal of Computational and Graphical Statistics*, 8(1):69–92.

Fox, J. and S. Weisberg
2019. *An R Companion to Applied Regression*, third edition. Thousand Oaks CA: Sage.

Gelman, A., J. Carlin, H. Stern, D. Duncan, A. Vehtari, and D. Rubin
2014. *Bayesian Data Analysis*, third edition. Chapman and Hall/CRC.

Gelman, A. and D. Rubin
1992. Inference from iterative simulation using multiple sequences. *Statistical Science*, 7(4):457–472.

Gelman, A., A. Vehtari, D. Simpson, C. C. Margossian, B. Carpenter, Y. Yao, L. Kennedy, J. Gabry, P.-C. Brkner, and M. Modrk
2020. Bayesian workflow.

Geman, S. and D. Geman
1984. Stochastic relaxation, Gibbs distributions, and the Bayesian restoration of images. *IEEE Transactions on Pattern Analysis and Machine Intelligence*, PAMI-6(6):721–741.

Hastings, W.
1970. Monte Carlo sampling methods using Markov chains and their applications. *Biometrika*, 57(1):97–109.

Hu, Y., S. Yuang, R. Hanner, J. Levin, and X. Lu
2018. Study of fish products in Metro Vancouver using DNA barcoding methods reveals fraudulent labeling. *Food Control*, 94:38–47.

Kim, B.-T. and M.-K. Lee
2018. Consumer preference for eco-labeled seafood in Korea. *Sustainability*, 10(9):3276.

Liu, Y., A. Gelman, and T. Zheng
2015. Simulation-efficient shortest probability intervals. *Statistical Computing*, 25:809–819.

Neal, R.
2011. MCMC using Hamiltonian dynamics. In *Handbook of Markov Chain Monte Carlo*. Chapman & Hall, CRC Press.

Newcombe, R. G.
1998. Two-sided confidence intervals for the single proportion: comparison of seven methods. *Statistics in Medicine*, 17(8):857–872.

Plummer, M.
2003. JAGS: A program for analysis of Bayesian graphical models using Gibbs sampling. *Proceedings of the 3rd International Workshop on Distributed Statistical Computing (DSC 2003)*, Pp. 1–10.

van Buuren, S. and K. Groothuis-Oudshoorn
2011. mice: Multivariate imputation by chained equations in R. *Journal of Statistical Software*, 45(3):1–67.

van de Schoot, R., S. Depaoli, R. King, B. Kramer, K. Märtens, M. Tadesse, M. Vannucci, A. Gelman, D. Veen, J. Willemsen, and C. Yau
2021. Bayesian statistics and modelling. *Nature Reviews*, 1:1.

Vehtari, A., J. Gabry, M. Magnusson, Y. Yao, P.-C. Brkner, T. Paananen, and A. Gelman
2023. loo: Efficient leave-one-out cross-validation and WAIC for Bayesian models. R package version 2.6.0.

Vehtari, A., A. Gelman, and J. Gabry
2017. Practical Bayesian model evaluation using leave-one-out cross-validation and WAIC. *Statistics and Computing*, 27:1413–1432.

Vehtari, A., A. Gelman, D. Simpson, B. Carpenter, and P.-C. Bürkner
2021. Rank-normalization, folding, and localization: An improved $\hat{R}$ for assessing convergence of MCMC (with discussion). *Bayesian Analysis*, 16(2):667–718.

Yao, Y., A. Vehtari, D. Simpson, and A. Gelman
2017. Using stacking to average Bayesian predictive distributions. *Bayesian Analysis*.
